# Improving genetics equity: identifying women eligible for genetic care services using mammography clinics in underserved areas as screening hubs

**DOI:** 10.1093/oncolo/oyaf113

**Published:** 2025-07-04

**Authors:** Darya Kizub, Rachel Bluebond, Sierra Green, Jessica Duckworth, Sreejesh Shanker, Autumn Vara, Banu Arun

**Affiliations:** Department of Breast Medical Oncology, The University of Texas MD Anderson Cancer Center, Houston, TX, USA; Department of Clinical Cancer Genetics, The University of Texas MD Anderson Cancer Center, Houston, TX, USA; Department of Clinical Cancer Genetics, The University of Texas MD Anderson Cancer Center, Houston, TX, USA; The Rose Center for Breast Health Excellence, Houston, TX, USA; Department of Clinical Cancer Genetics, The University of Texas MD Anderson Cancer Center, Houston, TX, USA; Department of Clinical Cancer Genetics, The University of Texas MD Anderson Cancer Center, Houston, TX, USA; Department of Breast Medical Oncology, The University of Texas MD Anderson Cancer Center, Houston, TX, USA; Department of Clinical Cancer Genetics, The University of Texas MD Anderson Cancer Center, Houston, TX, USA

**Keywords:** breast cancer, *BRCA*, hereditary cancer, genetic testing, genetic counseling, mammography, underserved, minority population

## Abstract

**Purpose:**

Fewer than 20% of underserved individuals undergo guideline-concordant hereditary breast and ovarian cancer (HBOC) genetic testing (GT). Our study aimed to determine the proportion of women eligible for HBOC GT using a cancer genetics risk assessment (CGRA) tool at breast cancer (BC) screening clinics in underserved communities and to describe the program’s impact.

**Methods:**

Participants were women who presented for BC screening at The Rose clinics, serving low-income underserved communities in southeast Texas, and completed the CGRA. High-risk individuals received bilingual educational materials and a saliva-based GT kit. Those with a pathogenic variant (PV) or a variant of uncertain significance (VUS) received telegenetic counseling and risk reduction resources.

**Results:**

A total of 501 women completed the CGRA, with 30.1% uninsured. 150 women were identified as eligible for GT, but only 14 (9.9%) completed GT (11 negative, 2 VUS, 1 PV in NF1). GT completion was significantly associated with being White, Native American/Alaskan Native, and Ashkenazi Jewish (*P* < .05). Hispanic, low-income, and uninsured individuals, or those with fewer relatives with cancer, were as likely to complete GT as others.

**Conclusions:**

We successfully identified underserved women at high risk of HBOC using CGRA, but the GT completion rate was low. However, the completion rate did not differ by Hispanic ethnicity, income, or insurance status, suggesting that financial navigation by our study coordinator, support from Spanish-language staff at The Rose clinics, and the use of Spanish-language educational materials and translation may have helped overcome some barriers.

**Implications for Practice**

Using a simple CGRA tool that collects personal and family cancer history, we screened 501 racially and ethnically diverse women for HBOC in underserved communities. We identified 150 women eligible for GT, who would have been missed without our intervention. The GT completion rate was low but notably, Hispanic, low-income, and uninsured individuals were just as likely to complete GT as others. Our study highlights the urgent need for patient-centered and convenient programs to improve the completion of guideline-concordant cancer GT, specifically tailored to underserved and minority patients, to bridge disparities in cancer outcomes.

## Introduction

Breast cancer (BC) is the most common cancer and the second leading cause of cancer-related death worldwide. Ovarian cancer (OC) ranks 11th in terms of incidence and is the fifth most common cause of cancer-related death in women.^1^ Hereditary breast and OC (HBOC) syndromes account for 5%-10% of BC and 15% of OC cases. Mutations in the genes *BRCA1* and *BRCA2* can increase the risk of BC in women to as high as 72% and that of OC risk to as high as 58% by age 70.^2^

Disparities in BC and OC outcomes are well documented. Black and Hispanic/Latino patients have higher BC mortality rates,^3,4^ and Black patients also have higher OC^5^ mortality rates. Mortality rates for both cancers are higher in patients of lower socioeconomic status and those with limited access to healthcare.^5,6^

The US Preventive Services Task Force (USPSTF) and National Comprehensive Cancer Network (NCCN) recommend HBOC genetic screening in women who are at risk for *BRCA 1*/*2* mutation based on their personal or family history of cancer.^7,8^ Positive results are used to inform family members about cancer risks, potentially preventing 23% of BC and 50% of OC cases attributable to HBOC.^9,10^ Despite high interest,^11,12^ rates of guideline-concordant HBOC generally range from 10%^13^ to more than 50%,^14^ but are usually under 20% for low-income,^15^ uninsured,^16^ and racial/ethnic minority patients.^17^

Low uptake of guideline-concordant GT includes low awareness about HBOC GT guidelines among primary care physicians,^18-22^ substandard family history collection,^23^ and insufficient access to genetic counseling (GC) in community practices.^24-26^ Nonetheless, identifying individuals at risk for HBOC is essential to reducing racial, ethnic, and socioeconomic disparities in cancer outcomes.^27^ Several clinical trials have aimed to identify individuals at risk for HBOC in primary care and improve access to GC and GT,^28,29^ including for racial and ethnic minority patients.^30,31^

In Texas in 2020, 60.9% of the population had a primary care doctor, and 77.7% of women had received a mammogram in the past 2 years,^32^ making breast imaging centers an ideal venue for patient outreach. Our group successfully validated an NCCN guideline-based cancer genetic risk assessment (CGRA) one-page survey tool that collects an individual’s basic sociodemographic information, and personal and family history of cancer by integrating it into the community breast imaging center’s workflow. The program successfully identified women at risk for HBOC. However, only 13.4% of patients made a pretest, in-person GT appointment, and only 10.2% completed GT.^33^

Telegenetics, or GC provided via telephone, is non-inferior to in-person GC^34,35^ and improves access to GT.^36^ Online education about HBOC without pretest GC, and with post-test GC for patients with a pathogenic variant (PV) or variant of uncertain significance (VUS) was equivalent to a combination of pre- and post-test counseling and improved GT completion.^37^

Our research aims to improve equity and access to genetic care by implementing our CGRA alongside novel strategies designed to improve GT completion at breast imaging centers serving minority and low-income communities. The primary objective of this study was to determine the proportion of women at The Rose clinics eligible for HBOC GT. Secondary objectives included establishing the proportion of eligible women who completed GT and evaluating factors associated with GT completion and patient-reported reasons for declining GT.

## Methods

### Program design and eligibility

Our study was a prospective feasibility study of women who presented for BC screening at The Rose Clinics, a nonprofit organization that provides breast imaging services to low-income communities in southeast Texas but does not provide any clinical genetics services. The program combined our CGRA tool,^33^ (Supplementary File 1), with strategies designed to reduce barriers to GT: remote GT, telegenetics, GC for patients with PV/VUS only, educational materials in English and Spanish, and financial assistance for GT.

### Patient recruitment and genetic care services

All women who presented to The Rose Clinics for screening mammography from February 1, 2020, to April 30, 2021, were eligible to participate. Due to the COVID-19 pandemic, our study coordinator was on-site only during the initial and latter months, working remotely in between. The study coordinator also provided education to mammography staff about the study.

Either the study coordinator or mammography staff at the front desk briefly explained the study to women presenting for mammography screening and asked if they were interested in participating. Potential participants were given educational brochures in either English or Spanish and directed to watch a 5-minute educational video about genetic services available in both languages on a tablet. The educational information was based on that provided to patients seeking genetic care services at The UT MD Anderson Cancer Center; it was not tailored for any specific population.

Participants were enrolled by the study coordinator in person or remotely via phone if they signed the informed consent and completed the CGRA. The CGRA collecting family cancer history is a simple, one-page, paper form designed for the participant to fill out on her own before or after her mammogram visit. The Rose staff and the study coordinator were available to answer any questions.

Most front-desk staff at The Rose who helped distribute the CGRA spoke Spanish, as many of the clinic’s patients are primarily Spanish-speaking. When participants filled out the Spanish-language CGRA, the study coordinator and genetic counselor used both Spanish and English using Spanish translation services as needed.

The CGRA was scored the same day or the next by the program coordinator in conjunction with our genetic counselor. After scoring, our study coordinator contacted the patient via phone, email, or both, based on the patient’s stated preference at enrollment. A minimum of 3 attempts were made to reach out to each patient to inform them about their CGRA results. If the patient was unavailable, the study coordinator left a voicemail. For those who could not be contacted, a certified letter with the CGRA results was sent to the address on file.

Participants who were successfully contacted were informed of their CGRA results. Those identified as high risk for HBOC and eligible for GT received a detailed explanation of their results and mailed bilingual educational materials developed by genetic counselors at MD Anderson. Participants agreeing to GT were sent the GT company Invitae’s, chosen for its superior patient financial assistance plan, saliva-based genetic testing (GT) kit. The project coordinator collected patient insurance information and submitted it to Invitae, as well as submitted financial documents for patient assistance for those who requested it.

For GT, we selected the comprehensive multigene hereditary breast and OC panel which included the following genes: *APC, ATM, AXIN2, BAP1, BARD1, BMPR1A, BRCA1, BRCA2, BRIP1, CDH1, CDK4, CDKN2A, CHEK2, CTNNA1, DICER1, EPCAM, FH, GREM1, HOXB13, KIT, MBD4, MEN1, MLH1, MSH2, MSH3, MSH6, MUTYH, NF1, NTHL1, PALB2, PDGFRA, PMS2, POLD1, POLE, PTEN, RAD51C, RAD51D, SDHA, SDHB, SDHC, SDHD, SMAD4, SMARCA4, STK11, TP53, TSC1, TSC2,* and *VHL.*

If the GT results showed a PV or a VUS, the genetic counselor called the participant to inform her and provide guidance on how to inform family members. This included a letter explaining the associated BC and OC risks and resources for cancer prevention and early detection. Patients with negative CGRA results were informed by phone. Our study did not provide additional assessments for patients with a negative result. Patients were encouraged to discuss their results with their primary care provider.

### Program implementation

**Figure 1.**
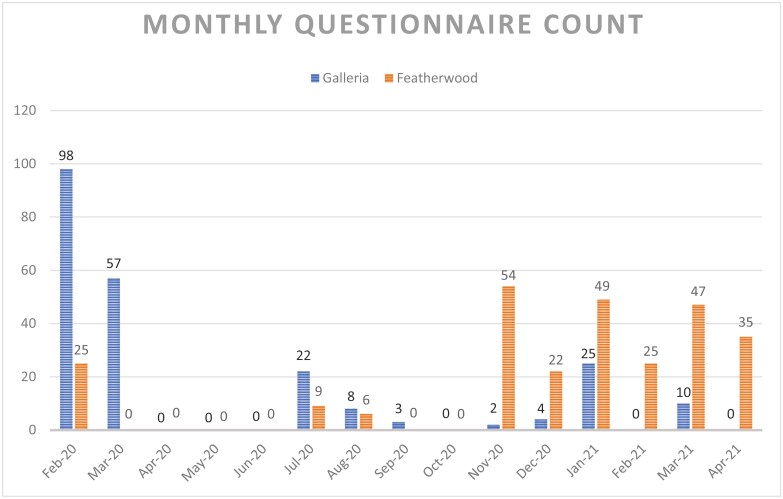
Monthly cancer genetics risk assessment surveys were completed at The Rose clinic sites in Houston.

Because of COVID-19 pandemic restrictions and limited staffing at The Rose, it was not possible to provide GC/GT education to the Rose staff, and patient accrual was uneven, dipping when the study coordinator was not on site (Figure 1). Because program reach was not formally assessed at the program design stage, it was not possible to determine how many participants were offered the program.

### Data collection and analyses

The CGRA included basic sociodemographic information (age, race, ethnicity, primary language, health insurance, and income), personal history and family history of BC, OC, pancreatic cancer, or prostate cancer.^34^ Participants who declined GT were asked to select a reason from a standard list based on data from prior studies.

Data were analyzed using descriptive statistics, with proportions for categorical variables and medians and standard deviations for continuous variables. The outcome dichotomous variable was set to either completing or not completing GT. Chi-squared or Fisher’s exact tests were used to test the association between categorical variables and the outcome, respectively. Logistic regression was used to calculate odds ratios for binary outcomes and linear regression for continuous ones, ie, the number of relatives with cancer; 95% confidence intervals are also reported. A *P*-value of < .05 was considered statistically significant. All statistical tests were two-sided. Statistical analyses were conducted with R version 4.2.2.

## Results

### Patient accrual

Because of the COVID-19 pandemic, our study coordinator could not be physically present onsite at either of The Rose clinics for most of the program. The clinics remained closed during the initial COVID-19 lockdown in Texas, and few patients were accrued during the Delta variant surge in the fall of 2021. Zero to 57 CGRAs were completed per clinic month during COVID-19 compared to 25-98 per month prior to the pandemic (Figure 1).

### Patient population

A total of 501 women completed the CGRA (Table 1). Analysis showed that the median age of participants was 52.0 years (SD 11.8; range, 21-79 years), with 52 (10.4%) aged 21-39 years. Among these women, 252 (50.3%) identified as White, 106 (21.2%) as Black/African American, 24 (4.8%) as Other, 13 (2.6%) as Asian, and 6 (1.2%) as Native American/Alaskan Native; 99 (19.8%) did not report their race. Ethnicity included 230 (45.9%) patients who identified as Hispanic/Latino and 7 (1.4%) as Ashkenazi Jewish. Spanish was reported as the primary language by 139 (27.7%) patients. Regarding health insurance, 151 (30.1%) patients were uninsured. Median annual salary was $45 000 (SD $97 563). Forty-nine (9.8%) patients had a salary of less than $19 000, 77 (15.4%) a salary of $20 000-$49 999, 43 (8.6%) a salary of $50 000-$74 999, and 63 (12.6%) had a salary of greater than $75 000; the remaining patients did not report their salary.

**Table 1.** Patient characteristics per the CGRA (*n* = 501).

Patient characteristic	Participants	(%)^a^
Age, years		
20-29	19	3.8%
30-39	33	6.6%
40-49	143	28.5%
50-59	139	27.7%
60-69	100	20.0%
70-99	38	7.6%
Unknown	29	5.8%
Race		
American Indian/Alaska Native	6	1.2%
Asian	13	2.6%
Native Hawaiian/Pacific Islander	1	0.2%
Black or African American	106	21.2%
White	252	50.3%
Other	24	4.8%
Unknown/not reported	105	21.0%
Ethnicity		
Hispanic/Latino	230	45.9%
Not Hispanic/Latino	150	29.9%
Unknown/not reported	121	24.2%
Ashkenazi Jewish descent		
Yes	7	1.4%
No	473	94.4%
Unknown/not reported	21	4.2%
Spanish-language primary	139	27.7%
No health insurance	151	30.1%
Annual income		
≤$19 999	49	9.8%
$20 000-$49 999	77	15.4%
$50 000-$74 999	43	8.6%
$75 000-$99 999	21	4.2%
$100 000-$149 999	25	5.0%
≥$150 000	17	3.4%
Unknown/not reported	269	53.7%
Personal history of BC	20	3.9%
Age at diagnosis, years (median)	51.8	
Triple-negative BC	5	1.0%
Bilateral BC	0	0.0%
History of OC	5	1.0%
Age at diagnosis, years (median)	45	
Family history of BC	189	37.7%
No. of relatives (*n* = 189)		0.0%
1	122	64.6%
2	46	24.3%
3	14	7.4%
>3	7	3.7%
Family history of OC	52	10.4%
Family history of pancreatic cancer	33	6.6%
No. of relatives (*n* = 33)		
1	29	87.9%
2	3	9.1%
3	1	3.0%
> 3	0	0.0%
Family history of prostate cancer	30	6.0%

^a^non. of patients (%) except where otherwise indicated.

Abbreviations: BC, breast cancer; CGRA, Cancer Genetics Risk Assessment; OC, ovarian cancer.

Twenty-five (4.9%) patients had a personal history of BC or OC, including 5 (1.0%) with a personal history of triple-negative BC. A family history of BC was reported by 189 (37.7%) patients, OC by 52 (10.4%), pancreatic cancer by 33 (6.6%), and prostate cancer by 30 (6.0%).

### Receipt of genetic care services

**Figure 2.**
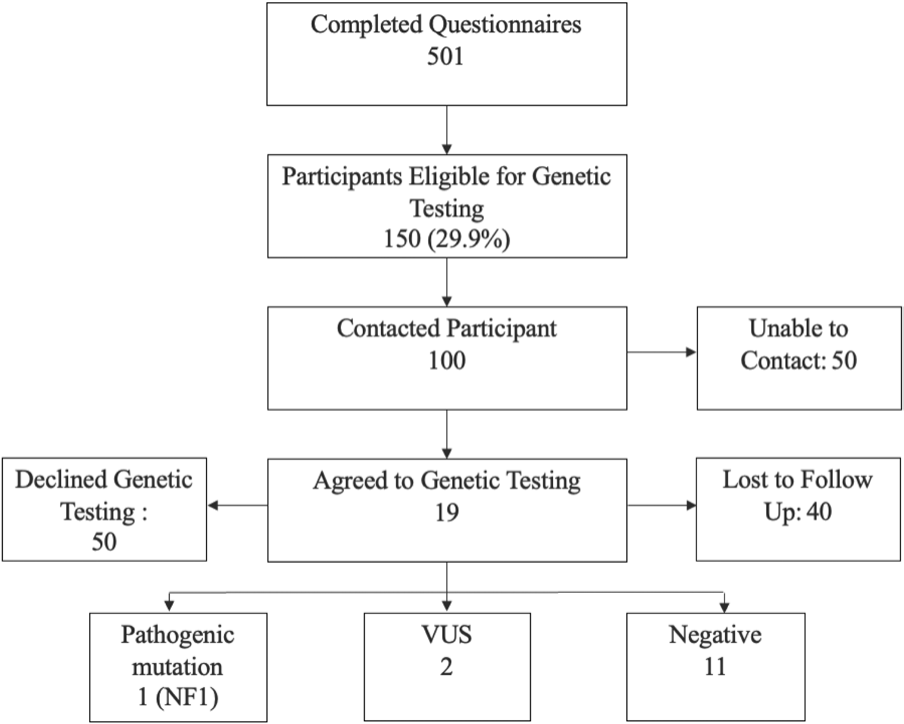
Eligibility for and receipt of genetic testing.

Out of 501 women, 150 (29.9%) were eligible for GT. Of these, 122 (81.3%) received genetic care educational materials. Contact was made with 100 women, who were then offered GT. Of these, 41 declined testing, 40 were lost to follow-up, and 19 agreed; 14 (9.3% of those eligible) returned the kits. 11 patients had negative GT results, 2 a VUS, and 1 a PV (*NF1*; Figure 2). All patients with PV or VUS received post-test GC.

### Factors influencing the completion of GT

GT completion was more likely in participants who were under 50 years old than in those 50 or older; those who spoke Spanish as their primary language compared to those who spoke English; and those who reported an income of less than $70 000/year (all *P* > .05) compared to $70 000 or more; and identified as White, Native American/Alaskan Native, or Ashkenazi Jewish (*P* < .05; in each case, compared to the rest of the participants) and less likely in those who were Black (vs. all others) or had a personal history of cancer (all *P* > .05; in each case, compared to the rest of the participants). GT completion was not associated with Hispanic/Latino ethnicity, the number of relatives with a history of cancer, or receipt of GC/GT education (Tables 2 and 3).

**Table 2.** Comparison of sociodemographic characteristics of patients who were eligible for and declined and completed GT.

Patient characteristic	Eligible for GT, n (%)		Declined GT, *n* (%)		Completed GT, n (%)		Completed GT/Eligible for GT
All	150	100%	41	100%	14	100%	9.3%
Age, years							
20-29	8	5.3%	2	4.9%	0	0.0%	0.0%
30-39	6	4.0%	1	2.4%	1	7.1%	16.7%
40-49	46	30.7%	8	19.5%	7	50.0%	15.2%
50-59	46	30.7%	16	39.0%	3	21.4%	6.5%
60-69	30	20.0%	8	19.5%	3	21.4%	10.0%
70-99	7	4.7%	3	7.3%	0	0.0%	0.0%
Race							
American Indian/Alaska Native	2	1.3%	0	0.0%	2	14.3%	100.0%
Asian	1	0.7%	0	0.0%	0	0.0%	0.0%
Native Hawaiian/Pacific Islander	1	0.7%	0	0.0%	0	0.0%	0.0%
Black or African American	33	22.0%	10	24.4%	1	7.1%	3.0%
White	86	57.3%	23	56.1%	12	85.7%	14.0%
Ethnicity							
Hispanic/Latino	68	45.3%	18	43.9%	7	50.0%	10.3%
Not Hispanic/Latino	40	26.7%	10	24.4%	5	35.7%	12.5%
Unknown/not reported	42	28.0%	13	31.7%	2	14.3%	4.8%
Ashkenazi Jewish descent	3	2.0%	0	0.0%	2	14.3%	66.7%
Spanish-speaking only	30	20.0%	17	41.5%	4	28.6%	13.3%
Insured	89	59.3%	24	58.5%	10	71.4%	11.2%
Uninsured	61	40.7%	12	29.3%	4	28.6%	6.6%
Annual income							
≤$19 999	14	9.3%	2	4.9%	3	21.4%	21.4%
$20 000-$49 999	30	20.0%	11	26.8%	6	42.9%	20.0%
$50 000-$74 999	17	11.3%	5	12.2%	2	14.3%	11.8%
$75 000-$99 999	6	4.0%	3	7.3%	0	0.0%	0.0%
$100 000-$149 999	8	5.3%	1	2.4%	0	0.0%	0.0%
≥$150 000							
Unknown/not reported	72	48.0%	18	43.9%	3	21.4%	4.2%
Personal cancer history							
BC	11	7.3%	2	4.9%	1	7.1%	9.1%
OC	5	3.3%	1	2.4%	0	0.0%	0.0%
Family cancer history							
BC	93	62.0%	26	63.4%	10	71.4%	10.8%
OC	52	34.7%	16	39.0%	7	50.0%	13.5%
Pancreatic cancer	33	22.0%	10	24.4%	5	35.7%	15.2%
Prostate cancer	27	18.0%	9	22.0%	0	0.0%	0.0%
Patient educational materials provided	122	81.3%	36	87.8%	14	100.0%	11.5%

Abbreviations: BC, breast cancer; GT, genetic testing; OC, ovarian cancer.

**Table 3.** Association of sociodemographic factors and cancer history with completion of gt in univariate analysis.

Sociodemographic Factor	Ref.	Odds ratio	95% Confidence Interval	*P*
Age < 50 years	Age ≥ 50 years	1.78	0.4-9.4	.49
White race	All others	5.2	1.1-23.3	.025
Black race	All others	0.25	0.03-1.99	.19
Hispanic/Latino ethnicity	All others	1.2	0.4-3.7	.71
Spanish as primary language	English as primary language	1.7	0.5-5.8	.40
Native American/Alaskan Native	All others	∞	1.9-∞	.0081
Ashkenazi Jewish heritage	All others	21.5	1.8-254.8	.015
Health insurance	Uninsured	1.3	0.4-4.5	.64
Income <$70 000/year	Income $70 000 or more	5.2	0.6-43.3	.13
Personal history of cancer	No personal history of cancer	0.6	0.7-4.7	.61
Number of relatives with a history of cancer		1.1	0.7-1.5	.75
Receipt of education about GC/GT	Did not receive education about GC/GT	∞	0.55-∞	.22

Abbreviations: GC, genetic counseling; GT, genetic testing.

### Self-reported reasons for not completing GT

Forty-one women declined GT for the following reasons: 6 (14.6%) each did not want to know the results or preferred to follow up in primary care, 3 (7.3%) each did not have enough time or had previously undergone GT, 2 (4.9%) were not interested or did not provide another reason, and 1 (2.4%) had only one distant relative. Additionally, 20 women (48.8%) could not be reached to find out the reason why GT was declined. Only one patient declined GT because of cost.

## Discussion

Our study successfully identified underserved women at risk of HBOC using our CGRA tool. This study was implemented at breast imaging clinics in racial/ethnic minority and low-income communities, despite the constraints of the COVID-19 pandemic.

In our study, 29.9% of women were eligible for GT compared to 1.9%-9% in other studies.^33,38-40^ Since we could not ensure the program was offered to everyone, women who perceived a higher risk of HBOC due to personal or family cancer history were more likely to enroll. Personal cancer risk perception has been linked to interest in genetic care services,^11,31^ GC referral,^41^ and GT completion,^42,43^ while family history of cancer showed mixed results.^44,45^ In our study, the number of relatives with a history of cancer was not linked to GT completion similar to findings where having a sibling with colorectal cancer did not increase screening behavior.^45^

Only 14 women (9.3%) eligible for GT completed it. A third could not be contacted, and 27.3% declined GT. Common reasons for declining included a preference for GT in primary care and not wanting to know the results. Less common reasons were lack of time and previous GT. Other studies noted a lack of interest and high costs as reasons for not pursuing HBOC GC/GT.^46^ Low-income patients were generally less likely to complete GT.^13^ However in our program, only one person declined due to cost, and participants with lower incomes tended to be more likely to complete GT, possibly due to financial assistance from Invitae and navigation support from our study coordinator.

Most prior studies using mammography as an entry point for HBOC genetic screening reported the proportion of patients eligible for genetic care, those who completed pretest GC, and those who completed GT, but not the total number eligible for GT.^38,47,48^ This is the first study to use breast imaging centers as entry points to improve access to HBOC GT for diverse low-income women via a CGRA. This approach included no requirement for pretest GC, post-test telegenetics only for patients with PVs or VUSs, non-tailored educational materials, and GT financial assistance.

In our prior study, 13.4% of eligible patients made an in-person pretest genetic counseling (GC) appointment, and 10.2% completed GT.^33^ Despite strategies to overcome barriers—such as education to raise awareness, financial navigation to address costs, and telemedicine to reduce appointment inconvenience—we could not improve the completion rate. The COVID-19 pandemic likely offset these benefits. The study coordinator’s absence on-site meant patients had to navigate multiple steps and contacts to complete GT. They had to answer a call about their cancer genetic risk assessment (CGRA) results, send insurance and ID information, receive a GT kit by mail, complete it, and mail it back.

Due to the pandemic, staff shortages, and busy schedules, our team could not train mammography staff on the importance of genetic care services. Non-tailored genetic education was not linked to GT completion. Different education methods, such as digital strategies (web-based applications, chatbots) tailored to uninsured/underinsured and minority populations, and pretest telegenetics, might improve GT uptake. Some of these strategies are being explored in clinical trials aimed at improving GT uptake in primary care and other settings, including for underserved and minority patients.^28-31^

In our study, a third of high-risk women were lost to follow-up and could only be notified of their high-risk status by certified letter. Studies have noted over 20% loss to follow-up in primary care, especially among patients with social vulnerability markers, such as nonWhite race, lack of insurance, younger age, and a primary language other than English. Healthcare-related factors, like poor relationships with the healthcare team and inconvenience, also contribute to loss of follow-up.^49^

Due to high loss to follow-up and low GT completion rates, our study team sought feedback from the study coordinator (SG) on improving the program. The coordinator identified several barriers: (1) lack of awareness among mammography staff about HBOC and GC/GT coupled with busy schedules leading to patients self-referring based on brochures rather than being offered the program; (2) significant loss to follow-up at each referral step due to phone contact requirements; (3) many patients were unavailable to talk during the day due to work. Based on these recommendations, future strategies to improve accrual and GT completion include: (1) standardized education for mammography staff on the importance of HBOC GC/GT; (2) scoring CGRA, providing results, and offering on-site GT sample collection if eligible; and (3) calling participants outside of business hours, including evenings and weekends.

The very low proportion of eligible patients who completed GT in our program, similar to that found in observational and interventional studies,^17,31,50^ highlights the critical unmet need for novel communication strategies to improve the utilization of evidence-based genetic care services in these underserved minority populations. While telegenetics was found to be non-inferior to GC in terms of GT completion, the rates of telephone GC completion were lower than in in-person GC, especially among minority patients.^51^ In another study, Hispanic/Latino women preferred printed materials and health coaching to web- or video-based education yet supported the use of a telephone-based CGRA combined with interactive health coaching and navigation as needed.^31^ A narrative review identified reasons for racial/ethnic disparities in *BRCA* GC/GT including lower awareness, cost, stress, distrust of the medical system, concerns about family and communication, and physician communication and referral. The researchers recommended multi-level interventions focused on education to increase awareness and risk perception, as well as improved communication within the medical team and with families.^52^

Studies have demonstrated high interest in BC risk assessment among minority low-income women^11^ but low rates of GT completion, especially in minority and underserved patients. A randomized controlled trial in a multi-ethnic, multi-lingual sample of women evaluated the effectiveness of a quick risk assessment tool with a patient-centered genetic risk report that was given to primary care physicians. This approach increased family cancer history discussions from 34.5% to 52.5%, risk discussions from 18.1% to 50.6%, high-risk genetics clinic visits from 4.1% to 18.8%, and GC/GT receipt from 4.1% to 20.8%. While these results indicate some success, there remains an unmet need for genetic care services in this population.^17^

Completion of GT was more likely in patients who identified as White, Ashkenazi Jewish, or Native American/Alaskan Native. In other studies, women who identified as White^13,51^ or Ashkenazi Jewish^53,54^ were more likely to complete HBOC GT. We did not find any studies that evaluated GC/GT in Native American/Alaskan Native patients.

Eligible Black women were less likely to complete GT in our study, but this finding did not reach statistical significance. This trend is similar to that of other studies,^16,55-57^ but the disparity was attenuated after adjusting for sociodemographic characteristics, including education, attitude about GT, and physician recommendation of GT.^58^ In Black women who were at risk for HBOC, higher self-efficacy was associated with a more positive attitude toward GC/GT,^59^ while higher medical mistrust was associated with less GC/GT engagement.^60^ Thus, interventions addressing trust and self-efficacy, including tailored education similar to that already developed^61^ or planned for development and testing in other studies,^62^ may be helpful to improve GT completion in Black women in future studies.

We did not find any associations between GT completion and Hispanic/Latino ethnicity or Spanish as the primary language, which contrasts with other studies. For example, fewer than 5% of primarily Hispanic/Latino women eligible for HBOC testing at an academic medical center received GT.^63^ Racial and ethnic minority patients and non-English-speaking patients were less likely to be referred to and complete GT,^52^ even with primary care interventions.^64^ Our results may be a result of assistance from Spanish-speaking staff at The Rose, our Spanish-language educational materials/videos, and the translation services that were used by our study coordinator and genetic counselor. Providing education in the participant’s preferred language and support tailored to the cultural and language preferences of underserved patients was a key strength of this study. Other studies have shown the effectiveness of tailored education for Latina women,^65-67^ and the potential use of community health workers in this setting.^68^

A systematic review of programs aimed at expanding access to genetic care services revealed varied results in the uptake of both genetic screening and counseling. However, the review noted insufficient descriptions of the programs to enable scalability, and the uptake of GT was not assessed. Therefore, our future programs will employ evidence-based implementation strategies and theoretical frameworks, along with process evaluations, to ensure the scalability of high-reach programs.^69^

The proportion of people eligible for genetic testing in our study was 29.9%, much higher than the 4% found in our previous study that used community mammography screening centers as entry points for GC/GT.^33^ Given the limited ability to educate all women presenting for mammography screening about GC/GT, we hypothesize that the high proportion of women eligible for GT was due to a greater likelihood that women with a strong family history and perceived higher cancer risk joined the study.

Over a third of the participants in our study lacked health insurance and could not undergo HBOC screening and referral through primary care. Of the 41 women who were eligible for and declined HBOC GT, only 3 had already undergone GT. Thus, the program successfully identified minority and low-income women at high HBOC risk who would have been missed by the healthcare system. This success is crucial for reducing disparities in HBOC GC/GT and ultimately reducing HBOC incidence and mortality.

### Limitations

The COVID-19 pandemic limited the ability of mammography center staff to engage and follow up with patients. An analysis of program implementation outcomes^70^ was not included in the program design but will be added to future similar studies. Consequently, data to inform program scale-up are limited, as we could not collect information about program reach (number of women offered CGRA), program adoption, or the proportion of women eligible to complete the CGRA who were offered study participation by The Rose staff or our study coordinator. Furthermore, while the majority of patients eligible for GT applied for financial assistance from Invitae, we did not collection information about the number of participants who applied and received it.

Our results are further limited by the high proportion of high-risk participants lost to follow-up. The exploration of factors associated with the completion of GT is constrained by the very small number and proportion of people who completed it and by missing data. For instance, fewer than 50% of the participants filled in annual salary information, about a fifth did not report race, and fewer than 10 participants identified as Native American/Alaskan Native or Ashkenazi Jewish. Therefore, results should be interpreted with caution. Due to the small sample size, we were not able to conduct a multivariate analysis to fully investigate predictors of testing; this should be explored in future larger studies.

Finally, while we asked patients to report and discuss the results of the CGRA and, if applicable, GT, with their primary care providers, we did not track adherence to this recommendation or the outcome. Linkages to primary care and resulting actions and recommendations from primary care providers would be important to explore in future studies.

## Conclusions

Our study successfully used a simple screening tool to identify underserved women at high risk of HBOC who had not previously undergone GT. We reduced barriers to GT by collaborating with community BC screening centers and utilizing remote testing and telegenetics. Only White and Native American/Alaskan Native race and Ashkenazi Jewish heritage were associated with GT completion. However, patients who identified as Hispanic were of low income, or were uninsured completed GT at the same rates as others, indicating that financial navigation and tailoring of services to language and culture may have reduced a few barriers. Results are limited by the small sample size. Given the small proportion of patients who completed testing, the next project phase will focus on improving convenience for patients and developing strategies to overcome patient and program-related barriers to GT completion.

## Supplementary Material

oyaf113_suppl_Supplementary_Material

## Data Availability

The CGRA tool and deidentified data underlying this article are available within the article and supplement information. Individual patient data includes personal health information (PHI) data and is protected by the HIPAA Privacy rule. Any other data will be shared on reasonable request to the corresponding author.
