# Supplementary material for: Improving genetics equity: identifying women eligible for genetic care services using mammography clinics in underserved areas as screening hubs
Source: Oncologist. 2025 Jul 4;30(7):oyaf113. doi: 10.1093/oncolo/oyaf113 (PMC12231590; doi:10.1093/oncolo/oyaf113)
Supplement: oyaf113_suppl_Supplementary_Material [file oyaf113_suppl_supplementary_material.pdf]

#35287256.0

Hereditary Cancer Risk Screening

Name:_______________________________________ Age (yrs)______ Preferred Language ☐ English ☐ Spanish

Address: ____

Phone #: ____________________________________ Email: ____________________________

Race ☐ White ☐ Black-Afro-American ☐ American Indian/ Alaska Native ☐ Asian ☐ Others: Specify ________________

Ethnicity ☐ Hispanic/Latin ☐ Non-Hispanic Annual Household Income: __________________________

Have YOU ever been diagnosed with any of the following?

Circle if YES*	Age(s) at diagnosis	Circle if No
Breast Cancer ☐<br>Was it triple negative: ☐ Yes ☐ No		No
Cancer in both breasts		No
Ovarian Cancer		No
Pancreatic Cancer		No
Endometrial Cancer		No
Colon Cancer		No
Other Cancer, specify:		

Has anyone in YOUR FAMILY ever been diagnosed with the following (maternal and paternal sides)?

Circle if YES*	Siblings/Children (who & age when diagnosed)<br>Ex: Sister 36 years<br>List all Members	Mother's side (who & age when diagnosed)<br>Ex: Aunt 44 years<br>List all Members	Father's side (who & age when diagnosed)<br>Ex: Uncle 65 years<br>List all members	Circle if No
Breast Cancer<br>Was it triple negative<br>☐ Yes ☐ No				No
Cancer in both breasts				No
Ovarian Cancer				No
Pancreatic Cancer				No
Endometrial Cancer				No
Colon Cancer				No
Metastatic prostate cancer				No
Male breast cancer				No
Other cancer, specify:				No

Are you Ashkenazi Jewish? ☐ Yes ☐ No

Have you or anyone in your family ever undergone genetic testing for cancer? ☐ Yes ☐ No ☐ Unsure

If yes, who? __________________________ What were the results? ☐ Positive ☐ Negative ☐ Unsure

If known specify what gene/s were tested: ____

*If you answered YES to any of the above questions, you may be contacted by our Patient Coordinator to discuss the benefits of genetic counseling for you and/or your family

For office use only

Patient appropriate for further risk assessment and/or genetic counseling ☐ Yes ☐ No
